# Harnessing Tolerogenic Histone Peptide Epitopes From Nucleosomes for Selective Down-Regulation of Pathogenic Autoimmune Response in Lupus (Past, Present, and Future)

**DOI:** 10.3389/fimmu.2021.629807

**Published:** 2021-04-14

**Authors:** Syamal K. Datta

**Affiliations:** Department of Medicine, Division of Rheumatology, Northwestern University Feinberg School of Medicine, Chicago, IL, United States

**Keywords:** autoimmunity, systemic lupus erythematosus, T regulatory cells, T suppressor cells, autoantigen specific tolerance, autoantigen derived peptide epitopes

## Abstract

Autoantigen-directed tolerance can be induced by certain nucleosomal histone peptide epitope/s in nanomolar dosage leading to sustained remission of disease in mice with *spontaneous* SLE. By contrast, lupus is *accelerated* by administration of intact (whole) histones, or whole nucleosomes in microparticles from apoptotic cells, or by post-translationally acetylated histone-peptides. Low-dose therapy with the histone-peptide epitopes simultaneously induces TGFβ and inhibits IL-6 production by DC *in vivo*, especially pDC, which then induce CD4+CD25+ Treg and CD8+ Treg cells that suppress pathogenic autoimmune response. Both types of induced Treg cells are FoxP3+ and act by producing TGFβ at close cell-to-cell range. No anaphylactic adverse reactions, or generalized immunosuppression have been detected in mice injected with the peptides, because the epitopes are derived from evolutionarily conserved histones in the chromatin; and the peptides are expressed in the thymus during ontogeny, and their native sequences have not been altered. The peptide-induced Treg cells can block severe lupus on adoptive transfer reducing inflammatory cell reaction and infiltration in the kidney. In *Humans*, similar potent Treg cells are generated by the histone peptide epitopes *in vitro* in *lupus patients’* PBMC, inhibiting anti-dsDNA autoantibody and interferon production. Furthermore, the same types of Treg cells are generated in lupus patients who are in very long-term remission (2-8 years) after undergoing autologous hematopoietic stem cell transplantation. These Treg cells are not found in lupus patients treated conventionally into clinical remission (SLEDAI of 0); and consequently they still harbor pathogenic autoimmune cells, causing subclinical damage. Although antigen-specific therapy with pinpoint accuracy is suitable for straight-forward organ-specific autoimmune diseases, Systemic Lupus is much more complex. The histone peptide epitopes have unique tolerogenic properties for inhibiting Innate immune cells (DC), T cells and B cell populations that are both antigen-specifically and cross-reactively involved in the pathogenic autoimmune response in lupus. The histone peptide tolerance is a natural and non-toxic therapy suitable for treating early lupus, and also maintaining lupus patients after toxic drug therapy. The experimental steps, challenges and possible solutions for successful therapy with these peptide epitopes are discussed in this highly focused review on Systemic Lupus.

## Introduction

This chronicle with historical perspective focuses first on the early steps of pathogenic autoantibody production in lupus, especially on the role of Th cells, and then how they can be regulated. In human lupus, it is now established that the main genetic risk loci for lupus susceptibility in GWAS are located in MHC class II and IRF5 regions, which respectively determine autoantigen presentation and associated activating cytokines production required to recruit autoreactive T helper (Th) cells (1–8). Genetic studies in families with rheumatic autoimmune diseases also support the initiating role of T cells in lupus (9), in addition to complement genes in the MHC locus (10). Importantly, augmented Th cell activity which is prevalent in lupus (11–13), can overcome the need for TLR abnormalities contributing to lupus (14). B cells and other professional APCs are activated to present autoantigens as the disease progresses. However, normally macrophages, such as, tingible body MΦ and DCs remain tolerogenic when handling dying (apoptotic) cells that can provide the autoantigens for lupus if mishandled, as described below (15–18). The professional APC become effectively activated *in vivo* to present these apoptotic autoantigens after the apoptotic cell derived DNA and/or RNA containing autoantigens are presented in IgG immune complexes (IC) that are bound by the APC to dually stimulate their TLR and FcγR (19, 20). Hence, Th cell mediated class-switched IgG autoantibodies specific for the DNA or RNA containing autoantigens have to be made first for IC formation activating the APC. Moreover, B cells become efficient antigen presenter to lupus Th cells that have been primed first by other APC, or if the B cells have developed high affinity receptors after undergoing somatic mutation and expansion with T_FH_ cell help in germinal centers (19, 21). However, high level expression of X-linked TLR7, due to incomplete X-chromosome inactivation (22), can contribute to lupus development early on, by independently activating DC and other APC, which in turn causes widespread T-cell activation (23, 24). To accomplish the above effect, striking studies have recently shown that IRF5 is first activated by TLR7 using the adaptor TASL, which interacts with SLC15A4, an amino acid transporter in endolysosome, to recruit IRF5 (25). The X-inked gene CXorf21-a encoding TASL and the gene for SLC15A4 were known to be associated with lupus susceptibility, as discussed in ref (26). Of course intrinsic defects in B cells and APC are critically important for lupus pathogenesis. With disease progression, other pathogenic players in T cell, B cell and unconventional APC populations evolve and are recruited to participate in amplifying the autoimmune inflammatory response, especially in extra-follicular sites, to cite a few (27–32), and reviewed elsewhere [Tsokos, 2020 #2492] (33, 34). Those pathogenic contributors might be kept in check by establishing regulatory mechanisms at the earliest steps of the disease, which is the focus of this review on Lupus, and this topic.

## Identifying and Cloning Pathogenic Anti-DSDNA Autoantibody-Inducing TH Cells of Lupus in Patients and Lupus-Prone Mice (Historical Perspective)

Step by step experiments and ensuing hypothesis based on their results at each stage led to cellular and molecular characterization of the pathogenic Th cells of lupus and how the Th cells become capable in helping pathogenic autoantibody production.

### Properties of Pathogenic Anti-DNA Autoantibodies

First of all, certain distinctive properties of pathogenic anti-DNA autoantibodies were crucial for isolating and characterizing the Th cells that specifically help them. The pathogenic anti-dsDNA autoantibodies that are deposited in kidneys with lupus nephritis have distinct features, as they are complement-fixing IgG in isotypes, with cationic charge, and clonally restricted by isoelectric focusing, and are able to cause glomerulonephritis *in vivo* (35–41). Moreover, their antigen combining V regions share recurrent idiotype and fine-specificity patterns for autoantigens (39, 42). Sequence analysis of the pathogenic autoantibodies confirmed their clonal expansion, as they shared V_H_ region CDR3 sequences containing numerous cationic residues generated by somatic mutation (43–45), a signature of Th cell drive. Contemporary studies had shown that immune complexes with cationic charge preferentially bind to anionic residues in glomerular basement membrane proteoglycans and collagen (46–48). It was shown later that glomerular binding of these “anti-DNA” antibodies could also be mediated *via* histones in nucleosomes bound *in situ* (49–52).

### Initial Studies to Find the Link Determining Cognate Interaction Between Autoimmune T and B Cells of Lupus

As described above, pathogenic anti-dsDNA antibodies in lupus are class-switched (35, 36) and clonally expanded (43, 44) suggesting a T helper cell dependent response, but it was mysterious up to 1980s and early 1990s how the Th cells actually helped Pathogenic anti-dsDNA autoantibody-producing B cells, because conventional Th cells do not recognize DNA.

In the first step, it was established that special autoimmune T helper (Th) cell subsets expanded the select population of pathogenic anti-dsDNA autoantibody producing B cells in mice with lupus (41, 53). The production of these pathogenic autoantibodies is also driven by select Th cells that are detectable in patients with active lupus nephritis (54–56).

In the next step, to define their antigenic specificity, the autoimmune Th cells were cloned from lupus prone mice, and also from patients with lupus nephritis (~ 800 clones). Prior to these studies, isolation of the pathogenic T helper (Th) cells of lupus was not done, because their antigenic specificities were unknown. However, using their special functional property of inducing the pathogenic variety of anti-dsDNA autoantibodies as a selection marker, the lupus nephritis-inducing Th cells were isolated. Only 12-15% of activated T cells in lupus patients and mouse models, could induce the production of pathogenic IgG anti-DNA autoantibodies (53–57). When administered into young pre-clinical stage lupus-prone mice, pathogenic autoantibody-inducing Th clones could rapidly induce immune-deposit glomerulonephritis (57, 58). Sequences of antigen-binding CDR loops of the TCRs of these pathogenic Th clones of lupus show recurrent motifs of anionic residues, indicating their selection by autoantigens with cationic residues (56, 57, 59). Indeed, a majority of such pathogenic Th clones produced IL-2 and IFN-γ in response to nucleosomes that contain histone peptides bearing cationic determinants, and nucleosome-specific T cells are detectable in pre-clinical stage lupus-prone mice before pathogenic autoantibodies are detectable in their serum (56, 60, 61). In addition, immunization of pre-clinical lupus mice, but not normal mice, with whole nucleosome particles induces accelerated lupus nephritis indicating the need for pre-existing autoimmune T and B cells in a lupus-prone background (60).

**Figure 1:**
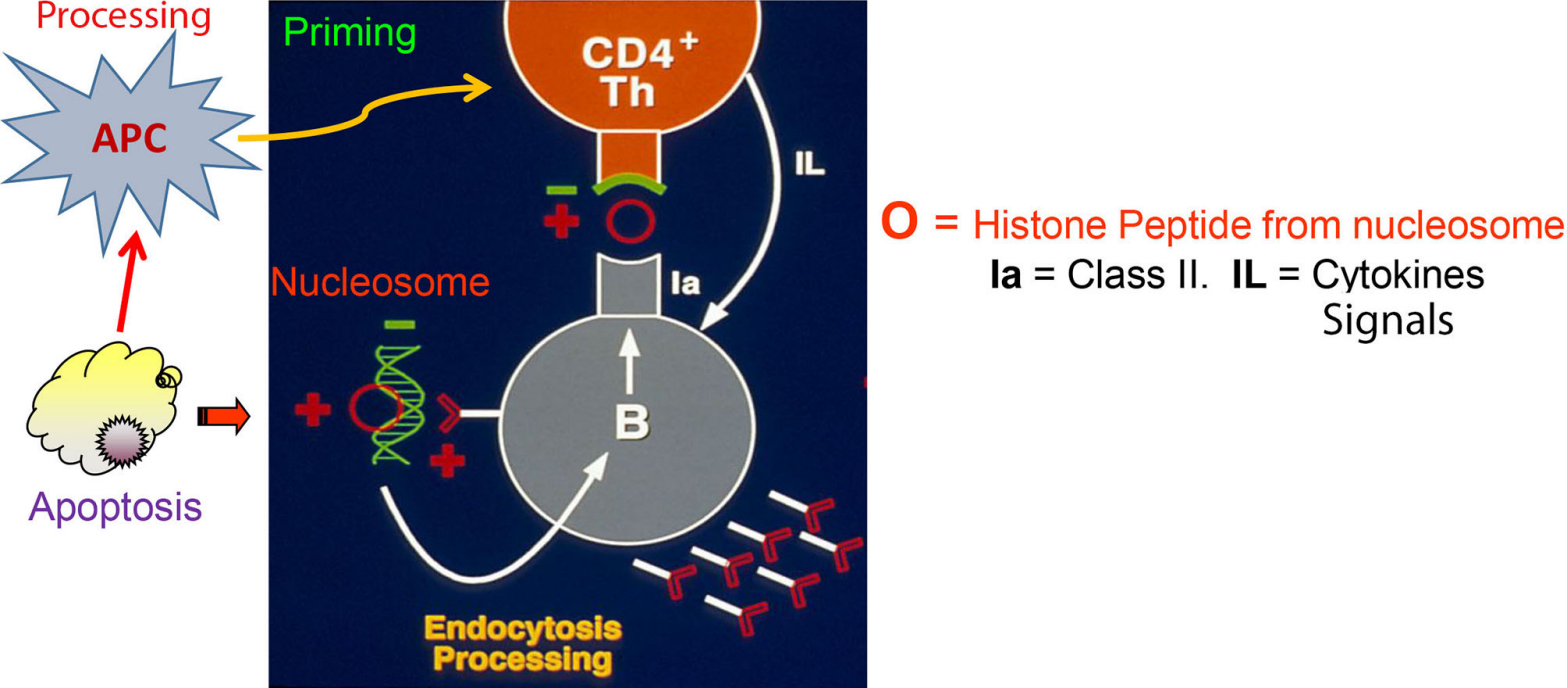
Autoimmune T and B cell interaction in lupus based on Nucleosome-derived autoantigens (based on work done from early 1980s through early1990s; references in the Text). Figure shows that Th cells that induce the production of pathogenic anti-DNA autoantibodies possess anionic residues in CDR3 of their TCRs (green). The lupus Th cells recognize peptides with reciprocal cationic charge (red), such as peptides from histones in nucleosomes presented by the pathogenic anti-DNA autoantibody producing B cells bearing BCRs with cationic residues generated by somatic mutations in CDR3 of their receptors (red). The pathogenic BCRs bind to anionic residues in DNA (green) that are complexed with cationic histones in nucleosomes, which are then endocytosed and processed for presentation to the interacting Th cells. Nucleosomes accumulate due to defective clearance of apoptotic cells in lupus, and are processed by activated APC to prime the pathogenic Th cells; a lupus-specific event initiated early in life. This figure is extensively modified from a figure in J Exp Med (60).

Thus the relevant autoantigens for pathogenic anti-dsDNA autoantibody inducing Th cells of lupus were discovered by an unbiased experimental approach, using pathogenic autoantibody inducing Th cells as *sensors* to detect the relevant autoantigen epitopes. This property provided the lupus Th cells the ability of “linked recognition” (62) for interaction with pathogenic anti-DNA autoantibody producing B cells (**Figure 1**). In this way, for the first time a true autoantigen for spontaneous SLE; namely endogenous nucleosomes from host’s apoptotic cells, and not some speculative component in microbes (63, 64), was found to be the real text book-like hapten- carrier link between the pathogenic Th and B cell in lupus for cognate interaction (57, 60),—and from that critical experimental step further studies led to identification of the histone peptide epitopes in nucleosomes recognized by those Th cells, and showed how to harness those particular epitopes for regulatory T cell induction for lupus therapy, as described below. All this was possible in 1980s and early 1990s by cloning the select population of pathogenic anti-dsDNA autoantibody-producing B cells, and then the special autoimmune T helper cells that drive such B cells in lupus. To emphasize again, despite the obstacle that the antigenic specificities of lupus T cells were then unknown, using the experimental steps described above, the pathogenic anti-dsDNA autoantibody-inducing T helper cells were cloned to define the structure and specificity of their receptor genes in human and murine lupus. To achieve the ultimate goal of understanding the cause, and designing a cure for spontaneous autoimmune diseases like lupus, it was essential at that time to identify the major autoantigen/s that drives the pathogenic autoimmune response. In lupus, DNA is a target antigen for autoantibodies but paradoxically immunization of mice with DNA does not cause lupus. The studies with the pathogenic autoantibody-inducing T helper clones in early 1990s led to the initial identification of one of the major immunogens that drives the pathogenic T helper cells of lupus (57, 60).

### Significance and Relevant Contemporary Studies by Others

Many studies soon followed that demonstrated or suggested mechanisms that could initiate or amplify the response of pathogenic Th cells to nucleosomal peptides in lupus. Briefly, in lupus, products from apoptotic cells accumulate and become immunogenic because, scavenging molecules in phagocytic cells, such as Marco and other scavenger receptors are functionally deficient in lupus, and so are complement components such as C1q, which facilitate phagocytosis of apoptotic cells without causing an immune response (65–68). Nucleosomes, HMGB1, DNA or RNA from apoptotic cell components not being disposed of properly, act as endogenous TLR ligands, stimulating cells of the innate and adaptive immune system (19, 23, 60, 69–74). For example, HMGB1 chromosomal protein from apoptotic cells that have not been removed properly, forms inflammatory complexes with other accumulating debris like DNA or nucleosomes particles, stimulating immune cells *via* TLR 2, TLR 4, and RAGE on the cell-surface, or TLR9 in the endosome/lysosome (74–76). Similarly, accumulating extra-cellular nucleosomes in micro particles containing DNA, or ribonucleoproteins containing RNA can stimulate cells of the innate immune system respectively by TLR9 or by TLR 7/8 (29, 31, 77, 78), thus augmenting autoantigen presentation to pathogenic Th cells by those activated APC. The case for TLR 9 is actually more complex, because in early stages, TLR9 actually protects against lupus (79), possibly by promoting tolerance in APC, B cells and helping Treg generation (80, 81). The other possible autoantigen-derived epitope with cationic charge, which could be recognized in a lupus B cell-linked fashion by the pathogenic autoantibody inducing Th cells possessing reciprocally charged anionic residues in their CDR3 region, would be derived from CDR3 region peptides of somatically mutated anti-DNA autoantibodies, as suggested (57, 60); and this possibility was independently demonstrated to be true by several laboratories (82–85). This issue is dealt in other contributions to this research topic.

### How Are the Many Types of Th Cells of Lupus Linked?

Th1, T_FH_, T_PH_, Gamma Delta Th, CD8 Th, CD4^-^CD8^-^ Th cells and more, participate in contributing to the pathogenic response in lupus. However, is it mainly Th1 → T_FH_ cells initiating/sustaining pathogenic autoantibody production; whereas the others evolve as amplifiers at extrafollicular inflammatory sites? First of all as mentioned above, only particular subclasses of IgG anti-DNA antibodies are more closely associated with a pathogenic potential in lupus patients and mice, and these pathogenic IgG antibodies belong to Th1-induced isotype classes. In lupus patients, Th1-induced anti-DNA IgG1antibodies are always elevated before the occurrence of renal relapse, and IgG1 plus IgG2 anti-DNA antibodies are found in patient’s renal eluates, whereas in lupus prone mice, murine Th1-induced IgG2a, IgG2b, and IgG3 anti-DNA are more frequently eluted from kidneys with active nephritis (39, 42, 86–88). In contrast to T_FH_ cells which conventionally produce IL-21; Th1 cytokine IFNγ not only mediates class switch for the nephritogenic isotypes, but Th1 derived IFNγ signal is also critical for autoantibody production by germinal center B cells (89, 90). Furthermore, many non-autoantigen specific, bystander T_FH_ cells expand as a secondary event with progression of disease, which could amplify (but not initiate) anti-DNA autoantibody production (91). Indeed, Ig class-switch recombination (CSR), during T and B cell cognate interaction, which is Th1 IFNγ cytokine dependent in lupus, may occur before the T_FH_ IL-21 driven expansion of autoantibody producing B cells in germinal center, which comes later (92). Moreover, *Th1-biased GC T_FH_ cells* have been reported (93), and another group reported that the differentiation and function of a *Th1-derived T_FH_1-like* cell population is driven by IL-12 signaling, which is important for differentiation of Th1 cells in the first place (94–96).

Therefore, *Th1 → T_FH_1 evolution/transition* is a possibility in pathogenic anti-DNA autoantibody production in lupus.

And then there are the potent T_PH_ cells with T_FH_ like phenotype but are CXCR5**^-^**; they help lupus B cells also by producing IL-21; and IL-10–producing CCR6^+^T cells populate lymph nodes of SLE patients. These Th cells probably evolve after receiving cytokine and other signals from activated B cells and other APC at extra-follicular sites, as the disease progresses (27, 97).

In addition, helper activity of CD8^+^ and CD4^-^/CD8^-^ αβ and γδ TCR^+^ Th cells, in pathogenic autoantibody production in human SLE has been reported (54, 55). The subset of T cells in humans that are CD4^-^/CD8^-^ and αβ TCR^+^ with pathogenic anti-DNA autoantibody-inducing ability in SLE is interesting, because such Th cells were considered to be unique to MRL-lpr mice with lupus. However, similar pathogenic autoantibody-inducing T cells with double negative phenotype that express “forbidden”, autoreactive T cell receptors were described in non-lpr lupus prone mice (41, 53, 98) and then in human lupus (54, 55). Although these double negative T cells might be secondary events in lupus compared to the CD4+ Th cells, they make an important contribution to pathogenesis of the disease. The CD4^-^/CD8^-^ and αβ TCR^+^ T cells also have important role in target organ inflammation (99, 100).

### Nucleosomal Peptide Autoepitopes Recognized by Pathogenic Th and B Cells of Lupus

In the next step, the critical peptide autoepitopes recognized by lupus nephritis-inducing Th cells were localized initially to be in the core histones of nucleosomes, at amino acid (aa) positions: 10-33 of H-2B, 16-39 and 71-94 of H4, and 85-102 of H3 (61). Altogether 154 overlapping 15-mer peptides spanning the entire length of all four core histones were tested to find the buried epitopes in nucleosomal histones that were recognized specifically autoimmune Th cells that cause lupus in mouse models. In addition, another dominant epitope was identified in position 22-42 of H1’, by mass spectrometry analysis of naturally processed peptides eluted from class II molecules of lupus B cell (APC) lines fed with chromatin (101). Remarkably, all these epitopes are located in regions of histones that contact with DNA in the nucleosome, and they are also targeted by autoantibodies from lupus B cells (B-cell epitopes), indicating that the epitopes could be protected from degradation during autoantigen processing and thus preferentially presented to the Th cells (61, 101–103). Surprisingly, the nucleosomal peptides have the features of “universal epitopes” (104), for instance, the peptide epitopes are promiscuously recognized by pathogenic Th cells derived from lupus-prone SNF_1_ mice (MHC I-A^d/q^) even when presented by APC bearing I-A molecules of all other mouse haplotypes, and human HLA-DR as well! Due to reciprocal charge interaction, the lupus TCRs probably contact the nucleosomal peptide-complexed with MHC promiscuously to sustain TCR signaling (105, 106). The promiscuity of lupus TCRs influences their selection in the thymus of lupus-prone mice and ability to generate Treg cells for tolerance spreading in the periphery, as described below (80, 107–109).

Nested Epitopes for CD8 T Cells. The tolerogenic nucleosomal peptide autoepitopes bind to MHC class II as described above, but CD8^+^ Treg cells were also induced by injection of the epitopes. Indeed algorithms showed, MHC class I-binding motifs were nested in their sequences, as described (108, 110). The rationale being that the relatively long chain peptides epitopes would be processed further by APC for cross-presentation to CD8 T cells (111). For an example, H4_71–94_ nucleosomal epitope has the nested CD8 sequence shown in bold letters TYTEHAKRKTVTAMDVVYALKRQG, and similarly individually distinct nested CD8 epitopes were detected in each of the longer peptide epitopes from the nucleosomes with CD4 Treg inducing ability, as detailed (108).

## Tolerance Therapy With Nucleosomal Peptide Epitopes

### Generation of Autoepitope Specific CD4 Treg and CD8 Treg Cell Subsets in Lupus by Low-Dose Tolerance Therapy With Nucleosomal Histone Peptides

(Experimental Steps of More Recent Publications in Mouse Models and Then in Human Lupus Are Described in Brief Below):

#### Studies in Lupus Prone Mouse Models

##### (a). Publication Title: “Very Low Dose Tolerance With Nucleosomal Peptides Controls Lupus and Induces Potent Regulatory T Cell Subsets”

**Figure 2:**
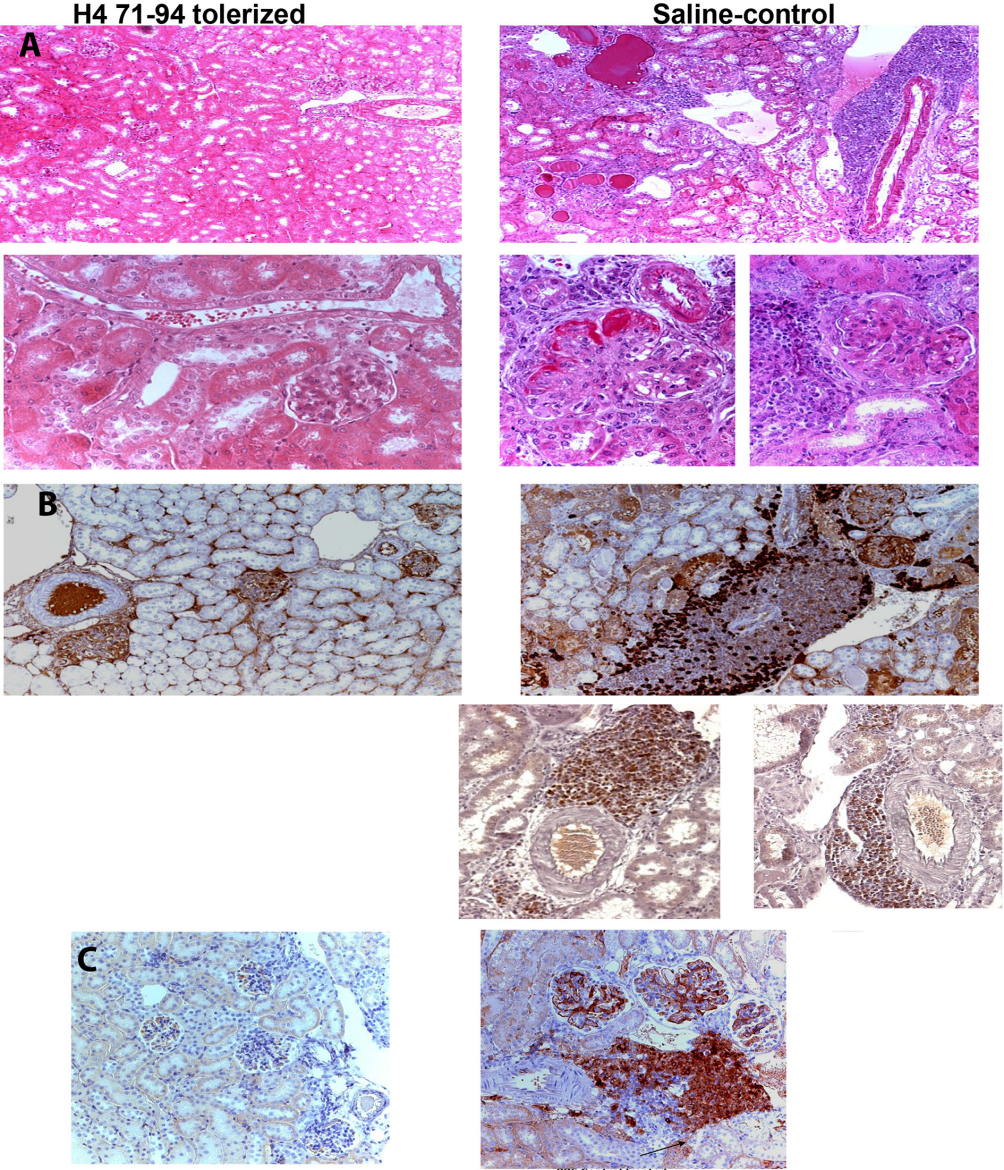
**(A)** Renal histology from lupus prone mice tolerized by histone peptide epitope (*left*) or age-matched saline-injected control mice (*right*). H&E staining; ×100 magnification shown in *Upper panels*. The saline control shows marked interstitial infiltrate of mononuclear cells with perivascular distribution, hyalinized and sclerotic glomeruli and tubules engorged with casts. *Lower panels* (original magnification, ×400) show in further detail the differences between mice that underwent peptide-epitope therapy (*left*) and control mice (*right*). Kidneys from the former group of mice show marked thickening of basement membranes and advanced sclerosis and crescent formation in glomeruli, and perivascular, interstitial infiltrates of mononuclear cells. **(B)** Immunohistochemistry (original magnification was ×200). Brown color shows positive staining for IgG deposits in glomeruli of lupus-prone mice, in both peptide-treated (*left upper panel*) and control groups (*right upper panel*). However, marked cellular infiltrates around blood vessels containing CD4^+^ T cells (on the *right, in upper panel*), CD8^+^ T cells (on *left in lower panel*), and CD138^+^ plasma cells (on *right side in lower panel*) were found only in kidneys of control mice, although both groups had IgG immune complex deposits. **(C)** Immunohistochemistry showing glomerular, and interstitial-perivascular infiltration of Th17 cells in control (PBS)-injected control lupus mice (*Right side*). This inflammatory cell infiltration was prevented in age-matched control mice by low-dose tolerance therapy with nucleosomal histone peptide epitope (*Left Panel*). Figure partially derived from J Immunol (80, 108), (Originally published in *The Journal of Immunology*. Kang H-K, Michaels MA, Berner BR, Datta SK. Very low-dose tolerance with nucleosomal peptides controls lupus and induces potent regulatory T cell subsets. *J Immunol* (2005) 174:3247-55. And Kang H-K, Liu M, Datta SK. Low-Dose Peptide Tolerance Therapy of Lupus Generates Plasmacytoid Dendritic Cells That Cause Expansion of Autoantigen-Specific Regulatory T Cells and Contraction of Inflammatory Th17 Cells *J Immunol* (2007) 178:7849-58. Copyright ^©^ [2005 and 2007] The American Association of Immunologists, Inc.).

**Figure 3:**
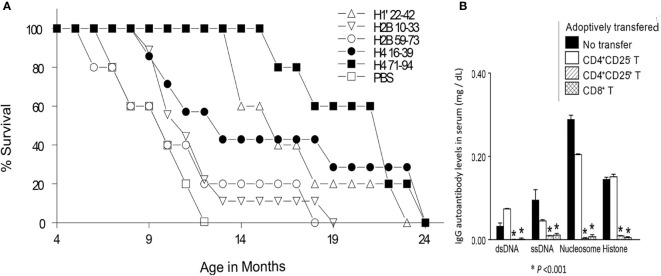
**(A)** Percent survival, of age-matched, lupus-diseased mice, injected subcutaneously with different nucleosomal histone peptides in low-doses, or with saline (PBS) every two week. **(B)** In a rigorous test for potency of suppression, Treg cells from H4_71-94_ peptide tolerized mice suppressed lupus acceleration upon adoptive transfer *in vivo*. Pre-clinical lupus-prone mice were *immunized* (not tolerized) by another histone peptide at 100 fold *higher doses* with *adjuvant* CFA, leading them to produce augmented levels of autoantibodies and develop severe nephritis rapidly, and this accelerated disease was suppressed by adoptive transfer of CD4+CD25+ Treg and CD8+Treg cells, but not CD4+CD25**^-^** effector T cells from H4_71–94_-treated tolerized mice. *Y-axis* values are for IgG autoantibody levels in serum of recipients (mg/dL). *P <0.001. Parts of this Figure are from J Immunol (80, 108). Experimental details are in those references. (Originally published in *The Journal of Immunology*. Kang H-K, Michaels MA, Berner BR, Datta SK. Very low-dose tolerance with nucleosomal peptides controls lupus and induces potent regulatory T cell subsets. *J Immunol* (2005) 174:3247-55. And Kang H-K, Liu M, Datta SK. Low-Dose Peptide Tolerance Therapy of Lupus Generates Plasmacytoid Dendritic Cells That Cause Expansion of Autoantigen-Specific Regulatory T Cells and Contraction of Inflammatory Th17 Cells *J Immunol* (2007) 178:7849-58. Copyright ^©^ [2005 and 2007] The American Association of Immunologists, Inc.)

The major autoepitopes for lupus nephritis-inducing Th cells were localized to H1’_22-42_, H3_85-102_, H4_16-39_ and H4_71-94_, as described above. These peptide epitopes stimulate both autoimmune Th cells and B cells. In lupus-prone mice, tolerance therapy at High doses (300μg I.V.) of the peptide epitopes halted the progression of established lupus nephritis. However, high-dose may not be suitable in humans. Therefore, low-dose tolerance therapy was developed with 300 fold lower doses by injecting lupus-prone mice with 1 µg nucleosomal histone peptide autoepitopes S.C. every 2 wk (108). This sub-nanomolar peptide therapy lowered autoantibody levels, blocked nephritis progression and markedly diminished inflammatory cell infiltration in kidneys, thus restoring normal life span. H4_71-94_ was the most effective autoepitope in this study. Low-dose tolerance therapy induced regulatory cell subsets of CD8^+^ suppressor Treg, and CD4^+^CD25^+^ Treg cells, which contained autoantigen-specific and cross-reactive autoantigen-directed Treg cells. The Treg cells suppressed IFN-γ production by pathogenic lupus Th cells in response to nucleosomal epitopes at up to 1:100 ratio of Treg or Treg : Th cells, and diminished autoantibody production *in vitro* by up to 90-100% by inhibiting nucleosome-stimulated T cell help to nuclear autoantigen-specific B cells. The induced CD4^+^CD25^+^ Treg and CD8^+^ Treg cells produced, and required TGF-β1 for immunosuppression; moreover, they effectively suppressed lupus autoimmunity upon adoptive transfer *in vivo*. For their suppressor function, the CD4^+^CD25^+^ Treg cells were partially cell contact-dependent, but CD8^+^ Treg cells were contact-independent. Thus, this work demonstrated that low-dose tolerance with the conserved histone autoepitopes durably ameliorates the regulatory defect in SLE by inducing TGF-β producing Treg cells, and without causing adverse side effects such as, generalized immunosuppression or allergic/anaphylactic reactions (**Figures 2** and **3**).

##### (b). Publication Title: “Low-Dose Peptide Tolerance Therapy of Lupus Generates Tolerogenic Plasmacytoid Dendritic Cells That Cause Expansion of Autoantigen-Specific Treg Cells Along With Contraction of Inflammatory Th1 and Th17 Cell Populations”

As noted above, low-dose tolerance of mice with lupus using just a single nucleosomal peptide epitope (H4_71-94_) could halt the progression of lupus nephritis by generating potent Treg cells that suppressed autoimmune T and B cells specific for a broad spectrum of nuclear autoantigens, and markedly inhibited inflammatory cell reaction and infiltration in kidneys. Next step was to determine how this therapy with only 0.36 nM of peptide injected subcutaneously (S.C.) every 2 weeks, induced *in vivo* TGFβ-producing CD8+Treg, and CD4+25+ Treg cell subsets containing regulatory cells that were autoantigen-specific, as established by the following approach (80). In order to track which APC had captured the histone peptide after tolerance therapy; DC, macrophages and B cells were isolated from local lymph nodes and spleens of lupus-prone mice injected with low-dose H4_71-94_ peptide S.C., and then those APC were tested for their ability to stimulate cognate H4_71-94_-specific T cell hybridomas in culture. The T cell hybridomas are highly sensitive and specific sensors detecting cognate peptide-MHC II on APCs presenting attomole concentration of the histone peptide. Only DC and B cells from spleen of histone peptide-injected mice stimulated the T hybridomas. Thus, during tolerance therapy, the subcutaneously injected H4_71-94_ peptide, which is highly soluble and charged, was rapidly absorbed systemically and captured by APC in the spleen. However, splenic DC, but not B cells or macrophages was responsible for the tolerogenic effect of the peptide therapy. Adoptive transfer of plasmacytoid DC or whole DC, but not B cells from the H4_71-94_ peptide treated mice suppressed responses of autoimmune T cells to nucleosome peptides up to 80% by Treg cells induced in the un-manipulated lupus mouse recipients, and blocked development of nephritis and autoantibody production in a lupus acceleration assay. The DC from the H4_71-94_ peptide injected mice expressed a tolerogenic phenotype upon capturing the S.C. injected H4_71-94_ peptide, expressing relatively low levels of CD80, CD86, CD40 and MHC class II. Compared to controls, the peptide epitope treated animal’s DC, especially plasmacytoid DC (pDC) produced increased amounts of TGFβ but decreased amount of IL-6 on stimulation by nucleosomes and other TLR-ligands, surprisingly the TLR-9 pathway was important for this tolerogenic effect (80). Moreover, these H4_71-94_ peptide-tolerized pDC ameliorated lupus autoimmune disease by simultaneously inducing/expanding contained autoantigen-specific and cross-reactive autoantigen-directed Treg, and suppressing effector Th1 and Th17 cells that infiltrate the kidneys causing lupus nephritis. As an aside, these studies initially showed that inflammatory Th17 infiltrate the kidneys of mice with lupus nephritis (80), which was then demonstrated also in human lupus nephritis (99, 112, 113).

Altogether, these studies early on showed the pathogenic importance of tubulo-interstitial region infiltration in lupus nephritis kidneys by various inflammatory cells in addition to monocyte/macrophages; such as extrafollicular germinal center like accumulation of CD4 and CD8 T cells, and B cells and plasma cells to set up residence in organized perivascular foci, as well as Th17 cells; and importantly, this infiltration was inhibited by the histone peptide epitope tolerance therapy resulting in its beneficial effect (80, 108) (**Figure 2**). As discussed below, similar to these therapeutic results, the role of locally active Treg cells migrating into the kidney and suppressing lupus nephritis has been recently demonstrated in other systems (114–116). Interestingly, **Figure 2B**, shows that IgG immune complex deposits were equally present in the kidneys in both peptide-treated and control lupus-prone mice, but interstitial infiltrates of interacting T and B cells and APC were prominent only in the control mice with severe nephritis. This observation is consistent with the demonstration that lupus B cells can contribute to nephritis even without autoantibody production, but just by autoantigen presentation and providing cytokine and other membrane signals to pathogenic Th cells (117); and that Belimumab has beneficial effect in patients with active lupus nephritis (118), which is surprising, but we now know that mature memory B cells also express BAFFR like immature transitional B cells (119). All these intricate pathogenic interactions were prevented by tolerance therapy with the histone peptide epitopes (**Figure 2**).

## Significance of Above Studies in Lupus-Prone Mice

### Cross-Reactive Recognition of Nuclear Autoantigens of Lupus and “Tolerance Spreading”

**Figure 4:**
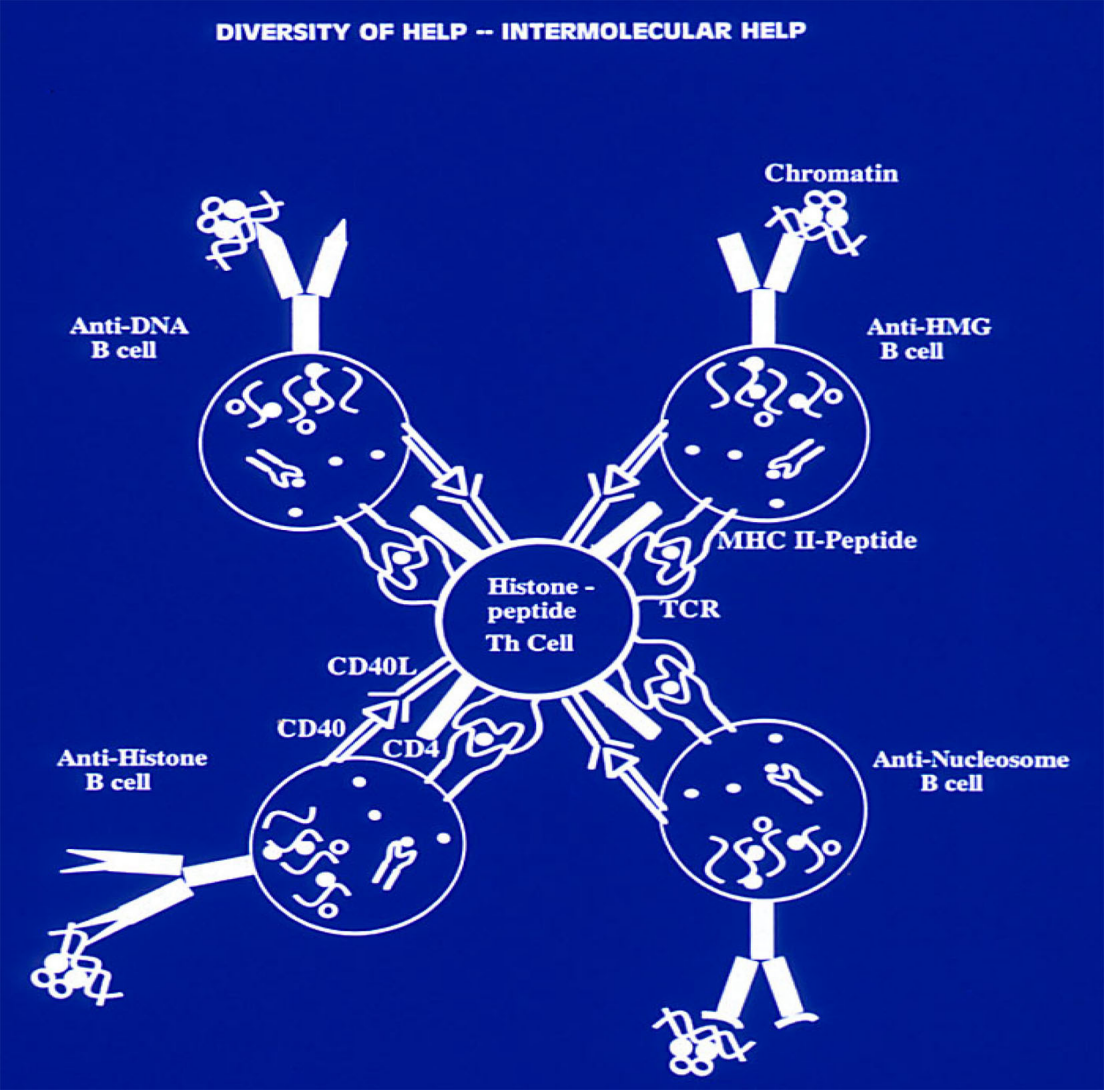
Production of a variety of anti-nuclear autoantibodies by inter-molecular T-cell help in SLE. A lupus Th cell with specificity for an individual nucleosomal histone peptide can help either a B cell specific for nucleosomes, or a B cell specific for dsDNA, or for ssDNA, or histone, or HMG, because each B cell can take up and process the whole chromatin particle by recognizing its own specific epitope in the chromatin, and then present to the Th clone its relevant histone peptide epitope derived from chromatin processing, which results in “inter-molecular help” This principle of linked “inter-molecular help” for a variety of B-cell epitopes in the complex chromatin particle would also apply to other Th cells of lupus which induce other pathogenic autoantibodies; and forms the basis for “Tolerance-Spreading” as described in the text. Modified from Ref (109, 120). (Originally published in *The Journal of Immunology*. Kaliyaperumal A, Michaels MA, Datta SK. Antigen-specific therapy of murine lupus nephritis using nucleosomal peptides: Tolerance spreading impairs pathogenic function of autoimmune T and B cells. *J Immunol* (1999) 162:5775-83. Copyright ^©^ [1999] The American Association of Immunologists, Inc.).

It is noteworthy that the immune response against nuclear autoantigens is inter-connected by cross-reactive recognition at the B cell level (38, 39, 42), and importantly at the Th cell level (61, 101, 102, 105, 109). Thus the same lupus Th clone can help either a B cell specific for nucleosomes, or a B cell specific for dsDNA, or for ssDNA, or histone, or HMG, because each B cell can take up and process the whole chromatin particle by recognizing its own specific epitope in the chromatin, and then present the Th clone’s relevant histone peptide epitope derived from chromatin processing, and that results in linked intermolecular help (56, 60, 120). Therefore, suppressing the Th cells of lupus could block spreading of response to multiple epitopes in chromatin (**Figure 4**). This hypothesis of “Tolerance Spreading” was experimentally supported as described above showing that progression of established lupus nephritis in the lupus-prone mouse models can be delayed, diminishing proteinuria and prolonging life by administering the nucleosomal peptide epitopes singly in high dose IV or low dose SC in tolerogenic regimens (80, 108, 109).

Indeed, the production of a variety of pathogenic autoantibodies to nuclear autoantigens was inhibited by tolerance therapy with any one of the epitopes. Due to promiscuous recognition described above, multiple autoimmune T cells with different TCRs can respond to the same peptide from a nucleosomal histone, and on the other hand, an individual autoimmune T cell can recognize multiple nucleosome-derived peptides that are distinct in sequence (61, 105). Therefore, when injected in a soluble tolerance-inducing form, in the absence of adjuvants, even one peptide epitope can tolerize autoimmune Th cells of diverse specificity for chromatin-derived autoantigens and conversely suppressing Th cells with specificty for one nuclear autoantigenic epitope deprives help for multiple autoimmune B cells of lupus. Thus, tolerance induced by any one of the dominant peptide epitopes can suppress autoimmune response to other nucleosome-derived pathogenic epitopes (“*Tolerance Spreading*”). Indeed, such “cross-reactive” suppression directed at the broad spectrum of pathogenic autoimmune response is more desirable in lupus therapy rather than pinpoint precision for antigen-specificity, which is the goal of some studies using modern techniques (121). Furthermore, the peptide epitopes are very effective in tolerance induction because, they are simultaneously recognized by autoimmune T and B cells, and they may inhibit autoimmune B cells and DC directly in lupus (80, 101, 108, 109).

Despite “Tolerance Spreading to other lupus autoepitopes”, the histone peptide therapy resulted in autoantigen-specific regulation because, pathogenic autoimmune responses in lupus-prone subjects were preferentially and selectively downregulated, without any suppression of immune responses to foreign antigens, as detailed in these References (80, 108, 109). Moreover, ability of the treated animals to survive environmental microbes/pathogens appeared to be intact and robust as compared to untreated controls and non-autoimmune “normal” mice housed in the same “dirty” mouse facilities (80, 108, 109). Finally, experiments showed that the Treg cells generated by the peptide epitope therapy suppressed T cell response and T cell helper activity specifically directed to the peptide autoepitopes, but not for a foreign antigen, such as Hen Egg Lysozyme (80, 108).

### Summary of Lessons Derived From Above Studies in Lupus-Prone Mice and Comparisons With Findings From Contemporary Literature

As described above, the nucleosomal histone peptide epitopes when administered in nanomolar doses (1µg) subcutaneously (S.C.) every 2 weeks or even every month to lupus-prone mice, are effective in delaying or even preventing nephritis. This dose is lower by almost 1000 fold compared to some other peptides being tried as therapeutic agents. Furthermore, a major histone peptide epitope, administered singly, can suppress lupus disease also *via* nasal tolerance (122, 123). The peptide epitopes are a constituent of nucleosome, a highly conserved, ubiquitous self-antigen produced during ongoing apoptosis in generative lymphoid organs and recognized by developing cells of the immune system bearing appropriate receptors. Therefore, anaphylactic reactions were not observed with these self-peptides when administered in close to 1000 lupus-prone mice for various studies. The histone peptide epitopes, administered S.C. in a very low doses, generate Treg cells that suppress by producing minute amounts of TGFβ that act in close range cell to cell interaction, rather than causing Th2 deviation with consequent allergic reactions seen in the case of therapy of other autoimmune diseases, such as, EAE/MS and diabetes in NOD mice using other peptides. The histone peptide epitopes induce stable Treg that are autoepitope-specific and cross-reactive autoantigen-directed Treg cells by simultaneously decreasing IL-6 and increasing TGF-β production by DCs, which consequently caused Smad-3 phosphorylation in the peptide epitope-specific auto-immune CD4+ Th cells, and the peptide tolerance therapy is effective even at an age when they manifest clinically active disease. A single dominant epitope such as H4_71-94_, is capable of inhibiting the diverse autoimmune process in lupus, because, potent and durable regulatory T cells (Treg) are generated by low-dose tolerance therapy mediating “tolerance spreading”. Both sets of regulatory T cells act *via* TGFβ in close range, and suppress autoimmune Th and B cells and other autoantigen presenting cells.

Although the phenomenon of low-dose tolerance was well known (124), most work since then have dealt with tolerance induction to foreign antigens in non-autoimmune subjects. However, the histone peptide induced low-dose tolerance was achieved in subjects with spontaneous SLE, whose immune system is already primed for autoimmune response against ubiquitous nucleosomal self-epitopes. The Autoantigen-specific and cross-reactive autoantigen-directed Treg cells generated by the peptide epitope therapy was effective even in the presence of complex lupus abnormalities, such as hyperactivity of lupus T and B cells and DC; particularly IFN-α producing pDC.

Unlike the peptide epitopes in low doses, whole (intact) histones worsen lupus (60) probably by binding to DNA or reciprocally charged molecules *in vivo* to make complex nucleosome like particle structures. Moreover, processing by APC of intact histones generates altered epitopes by post-translational modifications, such as acetylation or citrulination (125). Therefore, intact whole (complete) histones should not be used for tolerance induction.

Another group induced Treg cells by continuous infusion of a model laboratory antigen using hemagglutinin (HA)-specific TCR−Tg mouse system, and they also targeted the peptide to a surface receptor DEC-205 on DC, and administered considerable quantities of TGFβ *in vivo* (126). However, those approaches for therapy of diabetes were found to be deleterious (127). Moreover, *in vivo* administration of TGF−β as a drug in the presence of high IL−6 levels in lupus could induce pathogenic Th17 cells and T_FH_ cells, instead of generating Treg cells (128, 129).

## Recent Studies in Human Lupus Relevant to the Peptide Epitopes

Remarkably, pathogenic anti-DNA autoantibody inducing Th cells in human lupus recognized the same immunodominant histone peptide autoepitopes identified in murine lupus (61, 102), and those T cells in lupus patient’s PBMC respond by producing IFNγ. To reiterate, IFNγ-dependent IgG autoantibody subclasses cause lupus nephritis by fixing C’ and binding to inflammatory Fcγ receptors in pathogenic cells (39, 130). Furthermore, the peptide autoepitopes for Th cells of human lupus have the property of promiscuous HLA-DR binding, and as in lupus-prone mice, they are located in the native nucleosome at sites that contact with DNA, and they reside in histone regions that are also targeted by lupus B-cells (autoantibodies), thus being protected during antigen processing. Therefore, these immunodominant epitopes could probably be used as “universal” tolerogens in lupus patients despite their diversity of HLA alleles. The pathogenic role of nucleosome epitope-specific Th cells in human lupus have been confirmed by other laboratories (131, 132). Similar principles apply to other autoantigens in lupus, such as, Sm, RNP (133), but this review is focused on anti-DNA response whose pathogenic role in human lupus nephritis is well characterized. Remarkably, very recent approaches using latest technology to identify immunodominant epitopes for influenza hemagglutinin-specific memory T cells (134), showed results that are similar in outcome to the histone peptide approach performed two decades ago to identify the recurrent epitopes for pathogenic anti-DNA inducing memory T cells of lupus (61, 102).

### (a). Publication Title: “Regulatory T Cell (Treg) Subsets Return in Patients With Refractory Lupus Following Stem Cell Transplantation and TGF-β Producing CD8+ Regulatory Treg Cells (CD8^TGF-β^ Treg) Are Associated With Immunologic Remission of Lupus”

Unexpectedly, prolonged remission achieved by patients with refractory lupus after autologous hematopoietic stem cell transplantation (HSCT) have a different mechanistic basis than “clinical remission” in conventional drug-treated patients, who do not achieve true immunologic remission, although they have a Systemic Lupus Disease Activity Index (SLEDAI) of 0–2 (80% of the drug induced remission patients were at zero level). In patients with stem cell transplant induced remission, CD4+CD25^high^FoxP3+ Treg, and CD8+FoxP3+ Treg cells are generated, accompanied by almost total suppression of pathogenic T cells that respond to the histone peptide autoepitopes (135).

Detailed experiments in the above ref (135)., demonstrated that the post-transplant CD8 Treg cells suppressive activity was nucleosomal histone peptide-specific, as well as nonspecific, but directed to cross-reactive autoreactive and activated T cells. Both types of Treg cell’s suppressive activity was mainly TGF-β-dependent, but independent of cell-cell contact. The post-transplant patients’ CD8 Treg cells were stably FoxP3+ and they expressed markedly increased levels of CTLA-4, CD103, PD-1, PD-L1 and LAP, when compared to CD8 T cells from the same patients before undergoing transplantation. By contrast, the pre-transplant lupus patient’s CD8 T cells have cell-contact dependent helper activity for autoantibody production. The CD8 Treg found only in post-transplant patients are considerably more potent in suppressive activity compared to the CD4+CD25^high^ Treg cells that appear during clinical “remission” in lupus patients treated by conventional drugs, in whom autoimmune response of CD4 T cells to nucleosome-derived autoepitopes persists even during “clinical remission” (SLEDAI of zero). Therefore, autologous HSCT leads to the generation of a newly differentiated population of LAP^high^CD103^high^ CD8^TGF-^β Treg cells that maintain the lupus patients in “*true immunological* remission”, unlike patients with conventional drug therapy. Remarkably, very similar, highly potent CD8 Treg cells are also generated by low-dose nucleosomal peptide tolerance therapy that can prevent or treat lupus disease in mouse models of spontaneous SLE, as described above.

As stated, autoantibodies in lupus that belong to IFN-γ (Th1) dependent IgG subclasses fix complement and bind to activating FcγR on inflammatory cells to mediate pathogenicity. A CD4 T cell population in untreated lupus patients PBMC produces IFN-γ in response to histone peptide autoepitopes, and this autoimmune IFN-γ production response was almost completely suppressed in fresh PBMC from lupus patients in remission post-transplant. Removal of CD8 T cells (total) from the PBMC of post-transplant patients in remission, restored the IFN-γ response of CD4 T cells to nucleosomes and histone epitopes, much more strongly than removal of CD4+CD25^high^ cell subset enriched for Treg. Therefore, the latter subset probably cannot restore immunologic remission in conventionally treated lupus patients although they are increased in such patients after “clinical remission” (SLEDAI of 0–2).

The Post-transplant CD8 T cells suppressed by secreting mainly TGF-β and they expressed high levels of TGF-β latency-associated peptide (LAP), but they produced IL-10 to a much lesser extent; which is desirable because IL-10, by causing expansion of autoimmune B cells, is deleterious in lupus (136).

#### Significance of the Above Studies in Lupus Patients Transplanted With Autologous Stem Cells and Contemporary Relevant Studies by Others

The return of potent CD8^TGF^−β Treg cells after HSCT in refractory lupus patients, or after nucleosomal peptide epitope tolerance therapy in lupus-prone mice is an important biomarker for a state of true Immunologic Remission. These CD8+CD103+FoxP3+ TGFβ producing Treg are highly effective in controlling lupus, as shown in autologous stem cell transplant patients in remission above; and after corticosteroid pulse therapy induced remission in patients with lupus nephritis (Tsai YG et al. Plos One 2014, 9:e81344); as well as in murine models of lupus (108, 137, 138), and graft-versus-host lupus (138–140). And the above category of CD8+FoxP3+ TGFβ producing Treg that are highly effective in controlling lupus disease, are quite different from another variety of CD8+ Treg that are FoxP3-negative, cytotoxic and contact-dependent, and with varying surface phenotypes found in organ-specific autoimmune diseases (141–143). Those FoxP3**^-^**Ly49+(CD158e+ in humans)CD122^hi^Helios+CXCR5+ CD8 Treg cells were decreased as a percentage of total CD8 T cell population in lupus (144, 145), but such changes in proportion could be due to many reasons that cause shifts in various CD8 T cell subsets in lupus (146). Therefore, no cause and effect relationship of the latter CD8 Treg with spontaneous lupus disease in humans has been established yet. Anyway, target organ pathology in lupus nephritis, is inhibited by the TGFβ producing CD4^+^FoxP3^+^ Treg and CD8^+^FoxP3^+^ Treg cells induced by histone peptide epitope tolerance therapy (80, 108), or by targeted nanoparticle therapy (140) induced CD8+ Treg, which are quite different from the cytotoxic CD8 Treg (144, 145). Indeed, CD8+CD103+FoxP3+ TGFβ producing Treg cells, which maintain lupus patients in long term immunological remission after autologous bone marrow transplantation (135), or that induced in lupus patients’ PBMC by the histone peptide epitopes *in vitro* (147), have their highly effective suppressor counterparts in several models of autoimmune diseases including lupus (148–151). The role of locally active tissue-resident TGFβ producing Treg cells migrating into the kidney and its lymph nodes to suppress lupus nephritis pathogenesis, like those induced by histone peptide epitope therapy, has been recently demonstrated in other Treg inducing systems (114–116, 152).

### (b). Publication Title: “Major Pathogenic Steps in *Human* Lupus Can Be Effectively Suppressed by Nucleosomal Histone Peptide Epitope-Induced Regulatory Immunity”

As low-dose tolerance induced by the histone peptide epitopes effectively inhibited lupus disease in mouse models, the effect of the epitopes on lupus patients’ PBMC cultures was tested *in vitro*. As discussed above, the major Peptide Autoepitopes for nucleosome-specific T Cells of human lupus were identical in sequence to the peptide autoepitopes for pathogenic T cells of lupus-prone mice (61, 102), and they shared similar properties of promiscuous MHC class II binding and being B cell autoantibody epitopes as well. Thus the peptide epitopes could be effective tolerogens for inhibiting both autoimmune T and B cell populations in lupus patients with diverse HLA alleles (61, 102, 105, 147).

Indeed, in PBMC cultures from inactive lupus patients and healthy subjects, addition of the histone peptide epitopes induced CD4^+^CD25^high^FoxP3^+^ or CD4^+^CD45RA^+^FoxP3^low^ Treg cells, as well as CD8^+^CD25^+^FoxP3^+^ Treg cells with stable FoxP3 expression and suppressive activity (147). In the case of PBMC from patients with active lupus, dexamethasone or hydroxychloroquine were additionally needed for Treg-induction by the peptide epitopes in cultures. The peptide-induced regulatory T cells in lupus PBMC depended on TGFβ/ALK-5/pSmad 2/3 signaling, and TGF-β precursor LAP was expressed by those Treg cells, indicating that TGFβ production was responsible for their suppressive activity, and a positive feedback mechanism as well. The peptide epitope-induced Treg cells also inhibited type I IFN related gene expression in lupus PBMC. Expression of major members of Type I IFN genes themselves, as well as type I IFN *induced* genes (ISG) were markedly reduced by histone peptide epitopes in TLR9-stimulated PBMC of lupus patients. As stated above, pDCs in lupus are the main producers of Type I IFN upon stimulation by nuclear autoantigens complexed with anti-nuclear autoantibodies (7, 153, 154), and in lupus-prone mice, histone peptide epitopes act on pDC rendering them to become tolerogenic (80). Secondly, expression of 13 ISG genes, which have been reported to be upregulated in patients with active lupus (153, 155, 156) were also inhibited by the peptide epitopes. Moreover, the histone peptide Th cell epitopes, which were also shared by autoantibody producing B cell epitopes in lupus, could inhibit production of pathogenic autoantibodies by PBMC from *active* lupus patients as potently as an anti-IL6 antibody. Experimental details are in reference (147). Importantly, a mixture of the peptide epitopes (*cocktail)* was more effective in uniformly suppressing pathogenic activities in *Human* lupus PBMC cultures, as compared to single epitopes, because patients are heterogeneous in contrast to inbred lupus-prone mice. For example, suppression by a histone peptide cocktail #1 (C1), which is a mixture of H1^22-42^, H3^115-135^ and H4^16-39^ at a concentration of 1.5 μM for each peptide or histone peptide epitope cocktail #2 (C2), which is a mixture of H1^22-42^, H3^115-135^ and H4^16-39^ at a concentration of 4 μM of each peptide were very efficient in suppressing pathogenic autoantibody production and type I IFN related gene expression in lupus PBMC (147). Thus, low-dose histone peptide epitopes could durably inhibit pathogenic autoimmune response in human lupus by diverse pathways.

## Overall Clinical Significance and Summary

Generalized immunosuppression can control manifestations of active lupus, but despite their toxicity the drugs fail to achieve true immunological remission. Such drug therapies should be followed by autoantigen specific suppression of pathogenic autoimmune cells in lupus to prevent flares and continuing organ damage. In contrast to lupus patients, normal subjects have regulatory mechanisms including regulatory T cells that prevent abnormal pathogenic response to nuclear autoantigens from cells undergoing apoptosis routinely in the body (135, 147, 157).

The tolerogenic histone peptide epitopes have the potential for prophylactically repairing the functional deficiency of regulatory T cells in lupus (135, 147, 157, 158). The above studies in mouse models *in vivo*, and with lupus patient’s cells *in vitro*, showed that the peptide autoepitopes have the ability to bring about durable regulatory mechanisms; probably because of desirable properties mentioned above, and summarized here. The histone peptide epitopes are derived from nucleosomes of apoptotic cells produced daily in the body, which are cleared silently in the normal host without causing any immune response (17). Indeed large scale apoptosis occurs daily in generative organs, such as bone marrow and thymus and the products are used to “educate” the cells of developing immune system. The epitopes with their native sequences intact are called “unaltered peptide ligands (UPL)”; they are derived from nucleosomes of apoptotic cells that are naturally processed and displayed to developing lymphocytes during ontogeny (101, 107, 159, 160), and therefore, unlike artificially altered peptide ligands (APL), or post-translationally acetylated or citrullinated histone peptides, the unaltered histone peptide epitopes described here are not associated with anaphylactic/allergic reactions or worsening of lupus (80, 108, 109). In fact Treg cells are generated in the thymus, even in lupus-prone mice, in a natural response to the native unaltered histone peptide epitopes (107),. Only 1 µg (0.34 nanomolar) of the histone peptide epitope/s administered biweekly is effective in low-dose tolerance therapy of lupus-prone mice; that dosage would be around 0.2 to 2 mg range in humans with lupus. The histone peptides are rapidly absorbed systemically after S.C. injection, because they possess numerous charged residues making them highly soluble. As soon as they reach the lymphoid organs the peptides render APCs, especially pDC tolerogenic by inducing TGFβ and inhibiting IL-6, and consequently the peptide epitope presenting DC generate long-lasting Treg containing autoantigen-specific and cross-reactive autoantigen-directed Treg and Treg cells that suppress lupus (80, 108). Because the peptide epitopes operate by being taken up extremely rapidly by DC *in vivo* rendering them tolerogenic, short half life due to decay of the epitopes is not a problem. Moreover, the histone peptide therapy induced stable autoantigen-specific and cross-reactive autoantigen-directed regulatory or suppressive T cells generated *in vivo* are effective in suppressing disease upon transfer into lupus-prone mice (80, 108). Both MHC class II, and nested MHC class I binding determinants are present in the peptide epitope sequences so that they can genarate both CD4 Treg and CD8 Treg cells (80, 108). The epitopes are recognized by autoimmune T cells irrespective of the HLA type of lupus patients (102, 105, 135, 147, 159), similar to “universal epitopes” (104). Tolerance therapy with the histone peptide epitopes is effective even in mice with established lupus disease (80, 108, 109). The peptides can generate Treg in lupus patient’s PBMC even in the presence of other conventional maintenance medicines such as hydroxychloroquine or corticosteroids (147). The peptide autoepitopes from histones induce “linked tolerance” to other nuclear antigen autoepitopes recognized by pathogenic T and B cell of lupus (cross-reactive, “tolerance spreading”), but not to foreign antigens or other organ-derived autoantigens. In addition to generation of Treg cells, the peptides also exert tolerogenic effect directly on pathogenic lupus B cells and DC (80, 101, 147); suppressing autoantibody production irrespective of the degree of Treg induction (135, 147). Regulatory mechanisms against abnormal autoimmune response to nuclear autoantigens in asymptomatic subjects could be enforced by the relatively innocuous tolerance therapy with histone peptides (147), which suggests that apparently healthy relatives or ANA positive subjects at risk for developing lupus as predicted by GWAS bio-markers, could be protected prophylactically with these peptide epitopes.

Thus the peptide epitope therapy might be most suitable for maintaining lupus patients in true immunological remission after clinical remission has been induced by more toxic immunosuppressive agents. To summarize, unlike pinpoint antigen-specific therapy suitable for straight-forward organ-specific autoimmune diseases, the histone peptide epitopes directly or indirectly (through Treg cells they induce) suppress Innate immune cells (DC), T cells and B cells involved in the pathogenic autoimmune response in the complex systemic autoimmune disease, Lupus.

The histone peptide epitopes could also be used to develop sensitive diagnostic and/or prognostic tools (peptide-MHC tetramers) or assays (intracellular cytokine response) for tracking pathogenic Th cells that may appear prior to manifestation of the disease and elevation of autoantibodies. Indeed, understanding mechanism/s for generation of unusual and potent CD8+ Treg cells by the peptide therapy will be of therapeutic value in a broad spectrum of immune mediated diseases, and Immunologic Monitoring with the peptide epitopes may serve as biomarkers for true immunologic remission (supplementing conventional measures of clinical remission, such as, SLEDAI SLAM, BILAG).

## Future –Perspective, Problems that may Arise, and Possible Answers

Early phase clinical trials have shown promising outcome with autoantigen peptide therapy for inducing antigen-specific tolerance in several autoimmune diseases, such as Multiple Sclerosis and Type 1 Diabetes (161–167). These results are encouraging for clinical trials with histone peptides for lupus in the near future, but several distinct features of this lupus therapy need to be addressed to reach that goal. Unlike pinpoint antigen-specific therapy suitable for straight-forward organ-specific autoimmune diseases, the histone peptide epitopes have unique tolerogenic properties with broad autoreactivity-specific inhibitory effect. By rendering Innate immune cells (DC) tolerogenic, the histone peptides induce Treg cells that suppress T and B cell populations which are both antigen-specifically and cross-reactively involved in the pathogenic autoimmune response in the complex systemic autoimmune disease, Lupus:

### a) Low-Dose IL-2 and Corticosteroid Supplementation

Multiple laboratories have shown that histone peptide epitope/s or other peptide epitopes administered without IL-2 injection, can induce generation of effective Treg *in vivo*, which inhibit disease in various lupus-prone mice (80, 82–85, 108, 122, 168). Although lupus T cells are deficient in IL-2 production (169, 170), that situation is relative not absolute, as lupus patients do not succumb to recurrent infections found in IL-2 knockout Immunodeficiency. A possibility in the case of these peptide epitopes is that in low doses, they could transiently activate autoreactive T cells, which could then provide small amounts of IL-2 for generating the regulatory T cells. Indeed this proposed mechanism (171), has actually been demonstrated by a similar situation occurring in the thymus where IL-2 is produced by a small population of self-reactive CD4 single positive (CD4SP) thymocytes, which then stimulates Treg precursor cells to differentiate (172). Those regulatory T cells induced *in vivo* may then be sustained also by other signals such as from ICOS, or TNFR2 (Tseng WY et al. Proc Natnl Acad Sci USA 2019, 116:21666-21672) (173). Still in view of the benefits of low dose IL-2 therapy in all autoimmune diseases, whether deficient in IL-2 or not (129, 169, 170, 174–177); adjunct therapy with low-dose IL-2 will be beneficial in the peptide-epitope therapy of lupus, as stated in the theme of this Topic. Moreover, Low dose IL-2 and corticosteroids in maintenance dose, actually were necessary for the peptide epitopes’ induction of Treg cells in ACTIVE lupus patients’ PBMC *in vitro* (147). Indeed, corticosteroids themselves induce Treg cells by various mechanisms to some extent (Tsai YG et al. Plos One 2014, 9:e81344) (178, 179), and thus could potentiate the autoantigen-specific and cross-reactive autoantigen-directed Treg response by the peptide epitopes (147).

### b) Durable Treg Induction in the Midst of Inflammation; and Intrinsic Tolerogenic Properties of the Histone Peptide Epitopes

How can durable immunoregulatory mechanisms be established in the inflammatory environment of lupus? In lupus patients, tolerance therapy with histone peptide epitope would be optimal after inflammatory burden is reduced by drugs. Nevertheless, in animal models, the peptides alone are effective in ameliorating established lupus nephritis (80, 108, 109). The regulatory T cells are more stable in inflammatory environment because they were induced by the peptide epitopes *in vivo*, in contrast to Treg cells induced/expanded *in vitro*. Furthermore, dexamethasone or hydroxychloroquine in maintenance doses actually supported Treg-induction by the peptides in lupus patients’ PBMC cultures, indicating that drugs that counter the increased activity of IRF5 and TLR pathways in lupus APC would be of added benefit (147). The histone peptide epitopes also can directly regulate autoimmune B cells and DC in lupus, in addition to generating Treg cells (80, 101, 109); and indeed the peptides could suppress autoantibody production to baseline levels in lupus patient’s PBMC even before significantly increasing Treg cell numbers in culture (147). The select histone peptide epitopes, which are tolerogenic, can directly reduce IL-6 and increase TGFβ production by DC (80), a situation which renders the DC not only be able to induce Treg, but also become susceptible to suppression by Treg (180). This property of inducing TGFβ production and simultaneously decreasing IL-6 production by DC, especially pDC, in turn induces TGF-β signal (Smad-3 phosphorylation) in target auto-immune CD4+ T cells converting them to stable Treg cells; a property highly beneficial for lupus therapy (80, 147), also because T_FH_ cell differentiation is inhibited in germinal centers under such conditions (128).

Since apoptotic cells have immunosuppressive properties (17, 181); the unaltered histone peptide epitopes derived from apoptotic platform may have intrinsic tolerogenic property (80, 147). In very low doses, without immunostimulatory adjuvants, the histone peptide epitopes could possibly activate latent TGFβ, by inducing expression of the integrin αvβ8 in resting pDC, as shown in other systems (182).

### c) Peptide Delivery

Treg cells require continued antigen-specific stimulation from DC to maintain lineage stability and high affinity regulatory cell function (183, 184). In lupus-prone mice, regulatory T cells induced by the peptide epitopes are detectable up to six weeks after S.C. injection. Subcutaneous injection route works for the highly soluble and charged histone peptide epitopes which are very rapidly absorbed systemically (80). In humans many protein drugs, and mAb biologics, insulin, IVIg etc., are administered S.C. without causing local/systemic inflammatory response.

However, despite the promising beneficial effects in animal models of established lupus disease, there is always the possibility of adverse autoreactive response to the peptide epitopes in patients with lupus, although they might be selected at the earliest, pre-clinical stage of disease. Another issue is that peptide epitope cocktails in low doses were more effective than a single peptide epitope in suppressing lupus manifestations in human lupus PBMC, but cocktails may be more immunogenic when injected in the skin (147).

Therefore, peptide delivery should be considered in fail-safe tolerogenic vehicles, such as Nanoparticles (NP), which are described in detail by experts in this field in other articles as part of this research topic. Just as a brief synopsis, the peptide epitopes may be delivered within the nanoparticles, or administered around the same time, but separately from tolerogenic nanoparticles (185). There are many issues in choosing the right nanoparticles for such therapy, specifically for lupus; for instance, liposome derived NP can activate complement, and rapamycin containing NP may interfere with initial Treg generation (186, 187), although rapamycin is effective in maintaining Treg, once they are induced (188). It is noteworthy that injected nanoparticles might be nonspecifically immunosuppressive, like silica particles, by overloading the immune system’s APCs, which phagocytose and engorge themselves with any foreign particles (189, 190). Therefore, targeted nanoparticles designed to be directed against potentially autoreactive T cells are much more promising (140, 191), as addressed by articles from experts in this research topic.

Finally, emerging studies on epigenetic or metabolic mechanisms for Treg cell stability (192–194), and correcting other abnormalities in lupus T cells, such as, metabolic (12, 13), could be potentiated by utilizing the benefits of peptide epitope therapy, in the near future.

## Author Contributions

SD wrote and edited this review, and is accountable for the content of the work.

## Funding

This is a review of published work. Details of funding are in those publications cited in this review, and the work by the author’s group at that time had been supported partially by funding from National Institutes of Health (NIAID for 43 years, and NIAMS for 30 years), and Alliance for Lupus Research (TIL grant for 6 years).

## Conflict of Interest

The author declares that the research was conducted in the absence of any commercial or financial relationships that could be construed as a potential conflict of interest.
